# Exercise-Induced Oxidative Stress and the Effects of Antioxidant Intake from a Physiological Viewpoint

**DOI:** 10.3390/antiox7090119

**Published:** 2018-09-05

**Authors:** Takuji Kawamura, Isao Muraoka

**Affiliations:** Faculty of Sport Sciences, Waseda University, 2-579-15 Mikajima, Tokorozawa, Saitama 359-1192, Japan; imuraoka@waseda.jp

**Keywords:** exercise-induced oxidative stress, reactive oxygen species, free radicals, antioxidants, human trials, animal experiments, exercise performance, muscle damage

## Abstract

It is well established that the increase in reactive oxygen species (ROS) and free radicals production during exercise has both positive and negative physiological effects. Among them, the present review focuses on oxidative stress caused by acute exercise, mainly on evidence in healthy individuals. This review also summarizes findings on the determinants of exercise-induced oxidative stress and sources of free radical production. Moreover, we outline the effects of antioxidant supplementation on exercise-induced oxidative stress, which have been studied extensively. Finally, the following review briefly summarizes future tasks in the field of redox biology of exercise. In principle, this review covers findings for the whole body, and describes human trials and animal experiments separately.

## 1. Introduction

Humans and other aerobic organisms constantly produce free radicals as part of normal metabolic processes. Free radicals are defined as molecules or molecular fragments with one or multiple unpaired electrons in the atomic or molecular orbital. Free radicals derived from oxygen are called reactive oxygen species (ROS) [1]. However, ROS includes not only free radicals but also non-radicals, and are closely related to other free radical families, such as reactive nitrogen species [1]. Superoxides (O_2_^−^) and nitrogen monoxide (NO) are the primary free radicals that trigger a chain reaction of hydrogen peroxide (H_2_O_2_), hydroxyl radicals (OH^−^), peroxynitrite (ONOO^−^), and hypochlorous acid (HOCl) [1]. Although these free radicals have positive effects in immune reactions and cellular signaling, they are also known to have negative effects, such as oxidative damage of lipids, proteins, and nucleic acids [1]. As has been reported in previous studies, free radicals are constantly produced in various tissues (liver, kidneys, heart, skeletal muscle, etc.) at rest, inducing a certain level of oxidative damage in these tissues [2,3,4,5].

Organisms are equipped with antioxidant defense systems that protect cells from the toxic effects of free radicals. Antioxidant defense systems are divided into enzymatic antioxidants, such as superoxide dismutase (SOD), catalase, and glutathione peroxidase, and non-enzymatic antioxidants, such as vitamin C, vitamin E, glutathione, and bilirubin. These antioxidants play important roles in delaying or preventing oxidation of intracellular and extracellular biomolecules [6,7]. While the capacity of antioxidant systems is affected by intake of nutrients, such as vitamins and minerals, it is important to note that many mammals, with the exception of humans, can synthesize vitamin C in vivo [8]. This knowledge may be helpful in correctly evaluating the influence of supplementary intake and restriction of vitamin C on vitamin C concentration in tissues at rest and during exercise.

**Figure 1:**
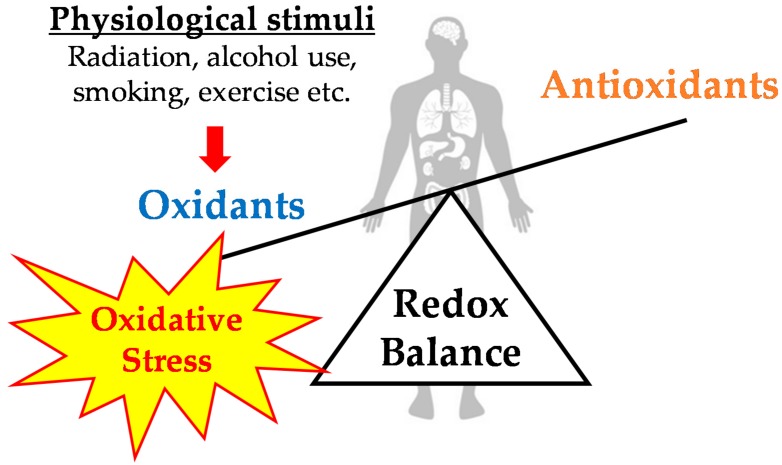
Redox balance and oxidative stress in living organisms.

As mentioned above, the oxidation-reduction (redox) balance is maintained in vivo by a complex regulatory mechanism; however, various physiological stimuli (i.e., radiation, alcohol use, smoking, and exercise) disturb this balance toward oxidation, thus inducing oxidative stress (Figure 1). Oxidative stress was initially defined as “a disturbance in the prooxidant-antioxidant balance in favor of prooxidants” [9], but was later redefined as “disturbance of the oxidation-reduction balance in favor of oxidants, leading to a disturbance in redox signaling and control and/or molecular damage” [10]. Previous studies have suggested that chronic oxidative stress is deeply involved in the onset or progression of various diseases such as diabetes, cancer, cardiovascular diseases, and neurological disorders, and may also play a role in the mechanisms of aging [11,12]. Thus, maintaining redox balance is pivotal for the survival and health of organisms. 

Powers and Jackson, in their own review in 2008, classified biomarkers that reflect in vivo oxidative stress into four broad categories [7]. The first method is to detect oxidants such as free radicals. Although most ROS and free radicals are highly reactive and have an extremely short half-life, which makes it difficult to measure them directly, they can be measured after first using exogenous molecules such as fluorescent probes or spin traps (i.e., 5,5-dimethyl-1-pyrroline-*N*-oxide and *N*-*tert*-butyl-α-phenylnitrone) to produce luminescence or to stabilize oxidants. However, as mentioned above, oxidative stress is determined by the redox balance, meaning that measurement of oxidants alone is not sufficient to accurately assess oxidative stress [7].

The second method is to measure antioxidant levels in tissues. Antioxidant levels (concentration or activity) vary according to tissue type, but can increase or decrease depending on the degree of exposure to oxidative stress. Antioxidant levels are useful markers of oxidative stress; however, nutritional status may affect the measurement results.

The third method is to measure oxidation products. Oxidation products include protein carbonyl (PC), which is a marker of protein oxidation; F_2_-isoprostanes and malondialdehyde (MDA), which are markers of lipid peroxidation; and 8-oxo-2′-deoxyguanosine (8-OHdG), which is a marker of deoxyribonucleic acid (DNA) oxidation. Measurement of these oxidation products is considered to be the most important aspect of oxidative stress assessment [7]. However, as these products exist only in trace amounts even during oxidative stress, they are often difficult to measure.

The fourth method is to measure the redox balance. The most frequently used redox marker is the reduced glutathione/oxidized glutathione (GSH/GSSG) ratio. Redox markers can be used to assess the redox balance with respect to both reduction and oxidation, thus making them extremely useful markers of oxidative stress. On the other hand, as with other methods, results can be affected by the techniques used to sample tissues or process tissue specimens [7]. 

As described above, as each oxidative stress marker has both advantages and disadvantages, there is currently no optimal marker for evaluating oxidative stress. Therefore, it was widely recommended to assess oxidative stress by measuring multiple oxidative stress markers [1,13]. 

During exercise, oxygen demand increases, particularly in skeletal muscle, causing a dramatic change in the blood flow to various organs. Furthermore, exercise-induced muscle damage promotes infiltration of phagocytes (i.e., neutrophils and macrophages) at the site of injury. These physiological changes that occur during acute exercise increase free radical production, leading to oxidative damage to biomolecules. Exercise-induced oxidative stress associated with increased free radical production has been studied for 40 years, since it was first reported by Dillard et al., in 1978 [14]. Recent developments in biochemical and molecular biological techniques have enabled observation of events at the cellular level, and have increasingly demonstrated that free radicals play at least some role in physiological adaptations after exercise training [15,16,17]. Therefore, free radicals generated by exercise are considered to have both positive and negative physiological effects.

The present review focuses on oxidative stress induced by acute exercise and outlines the effects of aerobic and anaerobic exercise on oxidative stress in organisms. This review also summarizes findings on the determinants of exercise-induced oxidative stress and sources of free radical production, about which much is still unknown. Finally, the present review briefly summarizes the effects of antioxidant supplementation on exercise-induced oxidative stress, which have been studied extensively. In principle, this review covers findings for the whole body, and describes human trials and animal experiments separately.

## 2. Exercise-Induced Oxidative Stress

### 2.1. Exercise-Induced Oxidative Stress: Human Trials

In 1978, Dillard et al., had subjects perform a 60-min cycle ergometer exercise at 50% $VO_{2}$ max intensity, and reported increased levels of expired pentane, an index of lipid peroxidation [14]. This study was the first human or animal study to suggest that exercise increases oxidative stress. Subsequently, Lovlin et al., in 1987 studied six young men who performed an incremental load exercise on a cycle ergometer to exhaustion, and found that the blood levels of thiobarbituric acid reactive substances (TBARS), another marker of lipid peroxidation, increased [18]. In a study by Gohil et al., in 1988, in which eight highly trained young men performed cycle ergometer exercise for 90 min at 65% $VO_{2}$ peak intensity, the levels of GSH, a non-enzymatic antioxidant, decreased, whereas the GSSG levels conversely increased [19].

These studies conducted in the late 1970s to 1980s generated a host of other studies on exercise-induced oxidative stress. To date, there has been a plethora of reports on the effects of acute aerobic exercise on oxidative stress markers [6,7,20,21,22]. However, the majority of these studies were conducted in healthy young men, and the subjects in each study seem different in training status. The most common types of exercise used in these studies are cycle ergometer or treadmill exercises in which the subjects typically engaged in maximal or submaximal exercise in a climate-controlled laboratory for 10–90 min. Some studies investigated the effects of eccentric contraction exercises, such as downhill exercises. Blood was by far the most commonly analyzed sample. Only a limited number of studies examined other biological samples, such as expired air, skeletal muscle, or urine. Oxidative stress assessments that use these biological samples often measure oxidation products of lipids, proteins, and DNA (i.e., MDA, PC, and 8-OHdG), as well as antioxidant levels and redox balance in tissues.

Studies conducted since the 1990s have reported that acute aerobic exercise increases the levels of markers of oxidative damage of lipids [23,24,25], proteins [25,26], and DNA [27,28]. Similarly, many studies have reported that acute aerobic exercise affects antioxidant levels and redox balance [25,26,29,30]. However, direct evidence showing that acute aerobic exercise increases free radicals and other antioxidants is extremely limited [31,32,33]. Thus, acute aerobic exercise is considered to induce oxidative stress in organisms. However, in a certain number of studies, exercise was observed to affect only some oxidative stress markers or none at all [26,34].

Exercise-induced oxidative stress can be triggered by aerobic exercise, as stated above, but has also been suggested to be triggered by anaerobic exercise. Marzatico et al., in 1997 had six young highly trained male athletes perform six sets of 150-m sprints and reported an increase in blood MDA levels [35]. In two separate studies in 2003, Groussard et al., had seven and eight young male subjects perform a 30-s full-strength pedaling exercise (the Wingate test) and subsequently detected an increase in blood free radical levels through electron spin resonance (ESR) spectroscopy [36,37]. These studies have also suggested that SOD activity in erythrocytes and levels of uric acid, vitamin C, vitamin E, and beta-carotene in blood considerably increase or decrease after exercise [33,34]. Several other articles have also demonstrated that the Wingate test causes changes in oxidative stress markers [38,39]. 

In addition to these studies using sprint exercises, other studies have investigated the effects of full-body resistance exercises (training muscle groups throughout the body using multiple types of resistance exercises) on oxidative stress markers. In a study in which 12 highly trained young men performed three sets of eight types of resistance exercises at 10 repetition maximum load, McBride et al., in 1998 found that the blood MDA levels increased [40]. This is one of several pieces of evidence demonstrating that full-body resistance exercises change the blood levels of oxidative stress markers [41,42]. Furthermore, local resistance exercises (defined here as exercises that train a specific muscle group using a single type of resistance exercise) also change the blood levels of oxidative stress markers [43,44,45]. Whereas all of these studies evaluated oxidative stress in blood, several other studies used muscle biopsies to confirm that local resistance exercises increase oxidative stress in skeletal muscle [46,47,48].

On the other hand, other studies found that resistance exercises do not affect oxidative stress levels in blood [49,50]. The discrepancy of these results may have been affected by training status. However, a study of individual changes in oxidative stress responses to eccentric exercise reported large inter-individual variability following acute eccentric knee extension exercise, even in subjects with the same training status [51]. This study further showed that in approximately one in three people, exercise-induced oxidative stress was either unexpected or negligible (response rate of 5% or lower) [51]. This result reasonably suggests that inconsistencies among studies in the results for both anaerobic and aerobic exercises can be attributed not only to training status but also to large inter-individual variability in exercise-induced oxidative stress.

### 2.2. Exercise-Induced Oxidative Stress: Animal Experiments

In an exercise in which rats swam to exhaustion while loaded with weights equal to 2% of their body weight, in 1979 Brady et al., found that the TBARS levels increased in the hind-limb skeletal muscle and livers of rats [52]. Likewise, in an incremental load test on rats exercised to exhaustion on an animal treadmill, in 1982 Davies et al., reported increased TBARS levels in the liver and hind-limb skeletal muscle [53]. This study is noteworthy in that it used ESR to determine that exhaustive exercise increases free radical levels in hind-limb skeletal muscle and the liver. This study was also the first to directly demonstrate that acute endurance exercise increases free radical production in tissues, thus serving as a major turning point in studies on exercise-induced oxidative stress. 

An overview of previous studies shows that many animal experiments, the majority of which were on rodents, have used an aerobic exercise protocol consisting of swimming or treadmill exercises. The animals used in these studies are often sedentary animals that have undergone a preliminary exercise regimen lasting several days before the main exercises. Whereas human studies examine blood samples, animal studies often examine skeletal muscle and other biological tissues. Oxidative stress is typically assessed by measuring oxidation products, antioxidant levels, and redox balance, similar to human studies.

Studies on laboratory animals have suggested that acute aerobic exercise increases oxidative stress levels not only in blood but also in tissues such as skeletal muscle and the brain, heart, lungs, liver, kidneys, and spleen [54,55,56,57,58,59]. It must be noted that, as Liu et al., demonstrated in 2000, the oxidative stress response to acute aerobic exercise is tissue specific [60]. In this study, post-exercise antioxidant levels (i.e., vitamin C, vitamin E, and glutathione) differed according to tissue type [60]. Thus, the oxidative stress response to acute aerobic exercise is believed to vary according to the type of tissue and the antioxidant levels in that tissue. 

As in human studies, anaerobic exercise is reported to trigger oxidative stress also in animal experiments. In an experiment by Alessio et al., in 1988, a 60-s treadmill exercise at a speed of 45 m/min drastically increased the MDA levels in the red vastus, white vastus and soleus muscles of rats [61]. This was the first study to demonstrate that anaerobic exercise, despite consuming less total oxygen than aerobic exercise, can also induce oxidative stress. Furthermore, Kayatekin et al., in 2002 had rats perform 15 sets of 30-s treadmill exercises at 35 m/min at a 5° slope with 10-s intervals, and reported an increase in TBARS level in the gastrocnemius muscle [62]. The increases in oxidative stress induced by these anaerobic exercises has also been observed in tissues other than skeletal muscle [63], which suggests that anaerobic exercise, like aerobic exercise, induces oxidative stress in various tissues. In addition to these findings, oxidative stress has also been induced in muscles in an exercise model consisting of muscle contractions triggered by electrical stimulation [64].

### 2.3. Advantages and Disadvantages of Human Trials and Animal Experiments

To summarize the advantages and disadvantages of human trials and animal experiments, studies on humans use physiological indices such as $VO_{2}$ and heart rate that enable precise setting of exercise intensity; however, assessment of oxidative stress in skeletal muscle or other tissues is not feasible owing to its high invasiveness. In contrast, animal experiments use very few types of exercise. Although not impossible methodologically, setting individualized exercise intensities is extremely difficult. However, in animal experiments, all kinds of biological tissues can be examined, and the effects of genetic and environmental factors can be eliminated to a greater extent than in human trials. Although human trials and animal experiments have their respective advantages and disadvantages, Veskoukis et al., have reported that exhaustive exercise at an intensity corresponding to 86% of maximum running speed resulted in approximately the same responses between humans and rats, in terms of oxidation products and antioxidant levels in blood plasma [65]. This suggests that results in rats sufficiently reflect the human oxidative stress response, at least in blood plasma. However, as these authors have concluded, elucidating the differences and similarities between experimental animals and humans is essential for appropriately interpreting the study results for both types of subjects. Specifically, further studies are required to examine the influence of exercise with relative intensity on oxidative stress responses in tissues other than blood. Toward this aim, it is essential to establish a methodology that can measure oxidative stress noninvasively under in vivo conditions (e.g., electron spin resonance computed tomography: ESR-CT [66]). 

## 3. Determinants of Exercise-Induced Oxidative Stress and Sources of Free Radical Production

### 3.1. Determinants of Exercise-Induced Oxidative Stress

Exercise-induced oxidative stress is considered to be greatly affected by exercise load. In 2003 Quindry et al., had young men perform exercises of varying intensities for the same duration, and found that the neutrophil production of superoxide, used in this study as an index of oxidative stress, increased only at intensities above the lactate threshold (LT) [67]. Furthermore, although the subjects in this study performed exercise at two different intensities for two different durations, both of which consumed the same total number of calories (4 or 60 min at intensities of LT + 10% or LT − 10%, respectively), changes in oxidative stress markers only occurred with exercises conducted at an intensity of LT + 10% [67]. This suggests that exercise intensity has a greater effect on changes in blood oxidative stress responses than on total caloric expenditure. Indeed, numerous animal and human studies have suggested that oxidative stress responses change in a manner dependent on exercise intensity [61,68].

However, in a study in 2007 in which young men and women performed a cycle ergometer exercise for 30, 60, or 120 min at a constant intensity (70% $VO_{2}$ max), Bloomer et al., reported that oxidative stress markers in blood (i.e., plasma PC levels) increased the most in the 120-min exercise [69]. Similar results have been reported in several studies on both humans and animals [70,71]. At a glance, these findings seem to contradict those of Quindry et al. [67]; however, exercise induces oxidative stress only when the intensity exceeds a certain threshold. Thus, it is conceivable that oxidative stress increases with the extension of exercise duration only when the above conditions are met. However, the physiological mechanism by which increased intensity or prolonged duration of exercise increases oxidative stress remains unknown. Ongoing research seeks to determine this mechanism based on differences in the production pathways of ROS and free radicals, as well as on differences in the responses of antioxidant defense systems to exercise.

As described earlier, oxidative stress is induced by a disruption in redox balance [10]. Therefore, studies are conducted from the perspective of oxidation promotion by exercise and from the perspective of antioxidation. One previous study has reported that a diet chronically deficient in antioxidants aggravates exercise-induced oxidative stress [72], whereas other studies have reported that antioxidant supplements inhibit exercise-induced oxidative stress [14,52]. In other words, a lack of antioxidants caused by factors such as an imbalanced diet can increase oxidative stress during exercise, whereas taking antioxidant supplements may inhibit exercise-induced oxidative stress. Therefore, for an ideal experiment, all subjects should be placed on identical diets in advance and should be prohibited from taking antioxidant supplements. In addition, as mentioned earlier, it must be noted that many mammals, excluding humans, can synthesize vitamin C in vivo.

It is also important that in vivo antioxidant levels can be improved not only through intake of exogenous antioxidants but also by exercise training. Many animal studies have reported that endurance exercise training increases antioxidant enzyme levels in skeletal muscle at rest [73,74]. Endurance exercise training has also been suggested to increase antioxidant enzyme levels at rest in the blood, heart, and liver [75,76]. These adaptation phenomena are also considered to be affected by the skeletal muscle site [77,78], muscle fiber type [79,80], training intensity and duration [77,78,81,82], and aging [83]. 

Thus, regular exercise may affect exercise-induced oxidative stress by increasing antioxidant levels. In fact, several studies have reported that long-term exercise training reduces oxidative stress following acute exercise [84,85]. Therefore, when conducting an experiment, the experimenter must have accurate knowledge of the training status of the human or animal subjects. An adaptation phenomenon called the “repeated bout effect” occurs in oxidative stress induced by eccentric contraction exercise, even after a single bout [86]. Therefore, repetitions of muscle-damaging exercise should be managed with measures such as fixed training intervals. Furthermore, it is important to pay attention to “regression to the mean” that occurs even in exercise without muscle damage; that is, when an individual who exhibited an extreme oxidative stress response after the first bout performs a second bout of the same exercise, the value of oxidative stress in the second bout is closer to the overall mean [87].

To summarize the above, exercise intensity and duration, nutritional intake, and training status are important factors that affect exercise-induced oxidative stress. In addition to these factors, aging, dehydration, hypoxia and sex have also been suggested to potentially affect exercise-induced oxidative stress [57,88,89,90,91]. Furthermore, a recent study has suggested that oxidative stress levels at rest affect the degree of exercise-induced oxidative stress [51]. However, evidence is limited about the effects of these factors on exercise-induced oxidative stress and the mechanism behind these effects, thus warranting further studies.

### 3.2. Sources of Free Radical Production during Exercise

An early study (from the 1970s) investigating the source of ROS and free radical production showed that 2–5% of oxygen consumed in the mitochondria at rest are converted to superoxides through one-electron reduction [92]. During exercise, oxygen consumption in active muscles is up to 100 times higher than at rest [93]. Thus, the primary source of ROS and free radical production was hypothesized to be the mitochondria. However, a later study provided a contradictory interpretation [94], whereas other studies have indicated that skeletal muscle mitochondria are not the primary source of ROS and free radical production during exercise [7,22]. 

Oxidative enzymes such as nicotinamide adenine dinucleotide phosphate (NADPH) oxidase and xanthine oxidase (XO) are considered to be involved in ROS and free radical production during exercise. NADPH oxidase is localized in the fascia, sarcoplasmic reticulum, T-tubules, and sarcolemma of skeletal muscle, and is believed to increase the production of superoxides by releasing electrons [1]. In contrast, XO is localized on the plasma membrane of skeletal muscle and is believed to produce superoxides by converting hypoxanthine into xanthine and uric acid [1]. In basic research using NADPH oxidase and XO inhibitors (i.e., apocynin or allopurinol), production of superoxides and increases in oxidation products in skeletal muscle were inhibited during exercise [95,96]. Although several potential sites of ROS and free radical production in skeletal muscle during exercise have been suggested, the primary source of production has not been definitively identified. Furthermore, as described earlier, exercise-induced oxidative stress can be triggered in other tissues as well, suggesting that tissues other than skeletal muscle should also be examined as potential sources of ROS and free radical production during exercise.

Exercise-induced muscle damage caused by downhill and resistance exercises, are known to generate a series of inflammatory responses in the damaged muscle tissue, thereby promoting infiltration of phagocytes such as neutrophils and macrophages [97,98]. Phagocytes infiltrating muscle tissue play an indispensable role in repairing and regenerating tissues, such as degrading proteins and eliminating cellular debris, and are understood to release a substantial amount of ROS and free radicals in the response process [99]. Although this production mechanism is yet to be fully determined, it has been shown to potentially involve the respiratory burst that occurs when phagocytosis is initiated [100]. Tidball has shown in 2005 that superoxide production is increased by NADPH oxidase during a respiratory burst, that the superoxides thus produced are promptly converted into hydrogen peroxide by SOD, and that this hydrogen peroxide is converted into strong oxidants such as hydroxyl radicals and hypochlorous acid by the Fenton reaction or the action of myeloperoxidase [100]. 

Thus, it has been demonstrated that exercise-induced muscle damage causes different time-course changes in general oxidative stress responses, mainly due to ROS and free radical production centered on phagocytes. Several prior studies have reported that free radical levels in blood and skeletal muscle increase immediately in exercises without muscle damage [31,32,33,53], which is in stark contrast to eccentric contraction exercise, in which an increase in free radical levels in blood is not observed until 72 h later [101]. Oxidative stress responses to eccentric contraction exercise are reported to be higher in individuals with sensitive responses in muscle damage markers (i.e., serum creatine kinase (CK)) [102]. These findings suggest that a certain delay occurs between eccentric contraction exercise and increased free radical production, compared with that for exercise centered on concentric contraction (e.g., flat running and cycle ergometer), in which free radical production increases at a relatively early stage.

Studies investigating the effects of differences in muscle contraction types on oxidative stress markers (i.e., oxidation products and antioxidant levels) have compared time-course change by taking samples multiple times after exercise [25,103]. The results of these studies, conducted by the same study group, showed that changes in oxidative stress markers in blood associated with exercise centered on concentric contraction reach a peak between 0 and 4 h following exercise, whereas the majority of these markers return to baseline values 6 h following exercise [25]. On the other hand, in eccentric contraction exercise, the marker values peak at 48−96 h after exercise and gradually return to baseline values by 7 days after exercise [103].

Few studies have observed long-term changes in oxidative stress markers until they return to baseline values. However, although the number of measurements taken was limited, many studies have reported that exercises centered on concentric contraction increase oxidative stress marker values at a relatively early stage (from immediately afterwards to several hours later) [14,18,19,52,53], whereas in eccentric contraction exercise, increases in oxidative stress marker values are delayed [43,101,102,104]. 

**Figure 2:**
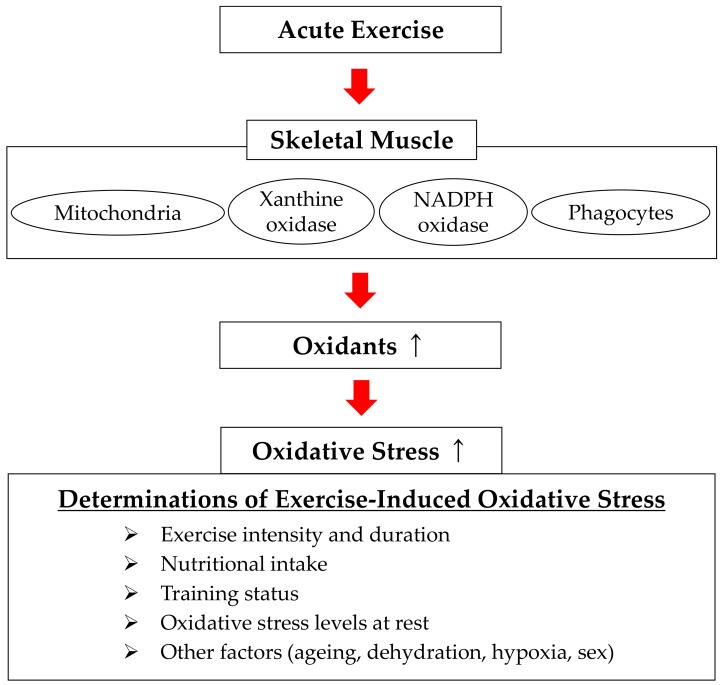
Determinants of exercise-induced oxidative stress and sources of free radical production. NADPH: nicotinamide adenine dinucleotide phosphate.

To summarize the above, a classification of acute exercises according to muscle contraction type shows that while oxidative stress occurs at a relatively early stage in exercises centered on concentric contraction, oxidative stress induced by eccentric contraction exercise occurs after a certain delay. In reality, definitively differentiating between the two types of exercises is difficult because exercise centered on concentric contraction can also induce muscle damage depending on the conditions of the exercise [105,106]. Nonetheless, the type of muscle contraction during exercise can have a major effect on the oxidative stress response induced by exercise, and therefore must be noted when conducting an experiment. Figure 2 shows the determinants of exercise-induced oxidative stress and the sources of free radical production. 

## 4. Effects of Antioxidant Intake on Exercise-Induced Oxidative Stress

### 4.1. Effects of Antioxidant Intake on Exercise-Induced Oxidative Stress: Human Trials

In an investigation in 1978 on the effects of taking vitamin E for 2 weeks (1200 IU/day) on oxidative stress induced by 60 min of cycle ergometer exercise at an intensity of 50% $VO_{2}$ max, Dillard et al. found that vitamin E inhibited the increase in the concentration of expired pentane, a marker of lipid peroxidation [14]. Sumida et al., similarly reported in 1989 that, in young men, an increase in blood MDA levels following exhaustive exercise on a cycle ergometer was inhibited by taking vitamin E for 4 weeks (300 mg/day) [107]. The effects of antioxidant intake on exercise-induced oxidative stress have also been examined in a great number of other studies. 

Previous studies have investigated the effects of a wide variety of antioxidant supplements, such as vitamin C, vitamin E, β-carotene, coenzyme Q_10_, α-lipoic acid, *N*-acetylcysteine (NAC), quercetin, resveratrol, and polyphenols, whereas other studies have also examined the effects of taking multiple such supplements. Depending on the study, these supplements are administered over the short term (i.e., single dose to several days) or the long term (i.e., 1 week to several months) at different times (before, during, or after exercise) and at different doses. Other aspects, such as subjects, exercise protocol (i.e., exercise type, intensity, and duration), biological samples analyzed, and methods of assessing oxidative stress, are nearly identical to general studies on exercise-induced oxidative stress.

Studies to date have reported that vitamin C [26,32], vitamin E [107,108], and a combination of these supplements [23,34] are effective in inhibiting exercise-induced oxidative stress. Other antioxidants have also been shown to be similarly effective [28,29,30,109]. 

The most notable physiological effect of inhibiting exercise-induced oxidative stress is improved exercise performance. In 1994 Reid et al., used NAC, a cysteine donor that increases the GSH of non-enzymatic antioxidants, to investigate the effects of free radicals on muscle fatigue, and found that intravenous infusion of NAC (150 mg/kg) inhibited tibialis anterior muscle fatigue induced by low-frequency electrical stimulation [110]. This study was the first human study to prove that free radicals can induce muscle fatigue and that supplementary intake of antioxidants can reverse it. In subsequent studies, intravenous NAC infusion inhibited diaphragm muscle fatigue [111] and improved performance in systemic endurance exercises [112,113]. 

The second most notable physiological effect of inhibiting exercise-induced oxidative stress is inhibition of muscle damage. As described earlier, large amounts of free radicals are produced primarily by phagocytes in the process of recovery from exercise-induced muscle damage [99]. The increase in free radicals following muscle injury has been suggested to cause secondary muscle damage directly or indirectly via oxidative damage of biomolecules or induction of inflammatory cytokines [100]. This is why numerous studies have been conducted to investigate the effects of antioxidant intake on muscle injury. 

Previous studies have reported that vitamin C [114], vitamin E [40,115], and a combination of these two vitamins [116] are effective for inhibiting muscle injury (i.e., blood CK and lactic acid dehydrogenase (LDH)). Other studies have also reported that antioxidant intake is effective against secondary oxidative stress [114,117] and delayed-onset muscle soreness [114,117,118]. 

It is important to note, however, that many studies have concluded that intake of these antioxidants does not affect exercise performance or muscle injury [119,120,121,122,123]. The inconsistencies between these results could be attributed to differences in the conditions of antioxidant intake (i.e., type, dose, duration, timing, etc.) and the exercise protocol. Recent studies have also shown that the efficacy of antioxidants is affected by inter-individual variability in redox state. Indeed, antioxidant intake has been shown to yield greater improvement in exercise performance in individuals with lower antioxidant levels at rest, whereas, conversely, antioxidant intake may reduce exercise performance in those with high antioxidant levels at rest [124]. Therefore, the selection of subjects who will take antioxidants may also have a major impact on the study results. Thus, while intake of antioxidants is considered to have a certain level of efficacy on the negative actions of exercise-induced oxidative stress, administration of antioxidants could also have a negative effect on individuals who already have an optimal redox state. In addition, it must be noted that in many previous studies, researchers have administered doses of antioxidants that largely exceeded the recommended dose.

### 4.2. Effects of Intake of Antioxidants on Exercise-Induced Oxidative Stress: Animal Experiments

Brady et al., reported in 1979 that a 4-week vitamin E-reinforced diet (50 IU/kg diet) inhibited increases in rat liver TBARS levels immediately after exhaustive swimming exercise [52]. The inhibitory effect of antioxidant intake on exercise-induced oxidative stress has also been observed in blood and other tissues such as skeletal muscle, the heart, and the kidneys [29,54,56,125]. 

Unlike studies on human subjects, many studies on laboratory animals have examined the effects of antioxidant intake on local muscle fatigue with electrical stimulation. Shindoh et al., reported in 1990 that intravenous infusion of NAC inhibited muscle fatigue in the diaphragms of rabbits [126]. This phenomenon was also observed in muscle groups in the lower limbs [127], suggesting that intake of antioxidant supplements can be effective for mitigating local muscle fatigue. 

In addition to studies on local muscle fatigue, many similar studies have investigated the effects of antioxidant intake on systemic endurance performance. Novelli et al., reported in 1990 that administering vitamin E intramuscularly substantially improved swimming performance in mice [128]. Similarly, numerous other studies have demonstrated that taking antioxidants improves endurance performance [129,130,131].

Like human studies, animal studies have also shown that taking antioxidants inhibits increases in the blood levels of muscle injury markers such as CK and LDH [132]. Kyparos et al., in 2011 used histological techniques (i.e., hematoxylin and eosin staining and toluidine blue staining) to directly demonstrate that antioxidant intake is effective for inhibiting post-exercise muscle injury [132]. 

As discussed above, numerous animal experiments have demonstrated that antioxidant intake is effective against exercise-induced oxidative stress and associated negative actions. However, it must be noted that many laboratory animals can synthesize vitamin C in vivo [8] and that many studies achieved repeated muscle contractions to the physiological limit by applying electrical stimulation.

### 4.3. Effects of Antioxidant Intake on Physiological Adaptation to Exercise Training

**Figure 3:**
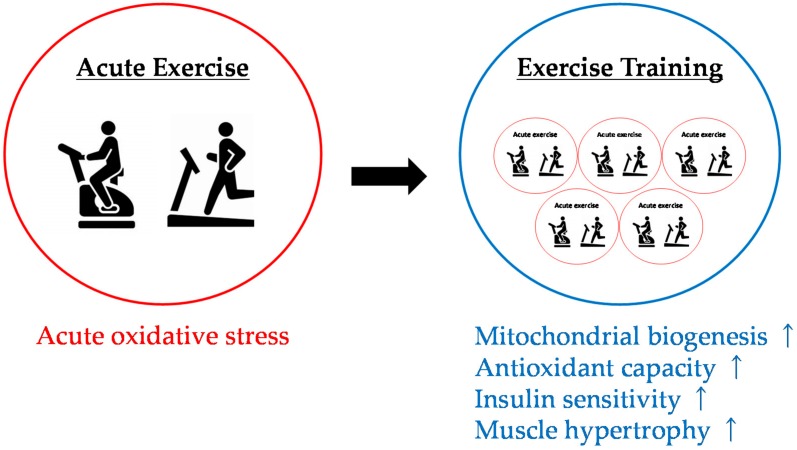
Reactive species and physiological adaptations to endurance training.

Gomez-Cabrera et al., reported in 2008 that long-term excessive administration of vitamin C inhibited skeletal muscle adaptation to endurance training and attenuated the improvement of endurance performance [133]. In addition, this study suggested that the adverse effects of vitamin C on exercise adaptation were caused by reduction of the expression of transcription factors involved in mitochondrial biogenesis, such as peroxisome proliferator-activated receptor gamma coactivator-1 alpha (PGC-1α), nuclear respiratory factor 1 (NRF-1), and mitochondrial transcription factor A (TFAM). Their study elicited great interest in whether antioxidant supplementation has a negative effect on the physiological adaptation of skeletal muscle to exercise training. To date, it has been suggested that long-term excessive administration of antioxidants inhibits redox-sensitive signaling pathways, and interferes with physiological adaptation such as mitochondrial biogenesis, improvement of antioxidant capacity, increased insulin sensitivity, and muscle hypertrophy [134,135,136] (Figure 3). Therefore, although numerous studies have presented counterevidence [137,138], long-term excessive intake of antioxidants should be avoided given that antioxidant supplementation has been shown to potentially inhibit the physiological adaptation of skeletal muscle to training. 

However, the majority of evidence indicating that ROS plays important roles as signaling molecule in physiological adaptation was obtained in in vitro experiments using myotube cells and in vivo experiments in which excessive amounts of antioxidants were administered. The former method frequently uses supraphysiological doses of ROS, meaning that estimating individual-level phenomenon based on these results is highly risky. In the latter method, it cannot be ruled out that the administered antioxidants may exert a variety of biological effects. Hence, it is considered extremely important to investigate the effects of ROS on exercise adaptation under physiological conditions in vivo and without antioxidants. Recently, a new approach trying to elucidate the role of ROS in exercise adaptation has been attempted using a stratification strategy based on oxidative stress responses to acute exercise [139]. Specifically, in this previous study, it was demonstrated that the degree of physiological adaptation (i.e., $VO_{2}$ max, time trial, and Wingate test) was greater in the “moderate” and “high” group than in the “low” exercise-induced oxidative stress group. This stratification strategy should be noted as a new research approach in the future.

## 5. Conclusions and Future Trends

The present review summarized the results of studies on the effects of acute exercise on oxidative stress in organisms, and the effects of antioxidant intake on exercise-induced oxidative stress obtained in human trials and animal experiments. The present review also summarized the determinants of exercise-induced oxidative stress, sources of free radical production during exercise, and time-course change in oxidative stress responses according to exercise type.

There is already a consensus that exercise increases the production of free radicals. However, free radicals are highly reactive and have extremely short half-lives, which makes them difficult to measure directly [1]. For this reason, previous studies have used numerous oxidative stress markers, including oxidation products, as indicators that reflect increases in free radicals. These markers of oxidative stress all have advantages and disadvantages, which is why oxidative stress should be assessed by measuring multiple markers [1,13]. Ultimately, this makes exercise-induced oxidative stress more difficult to understand. Furthermore, it is currently unknown whether changes in oxidative stress markers associated with exercise actually represent major deviations from optimal ranges, from a physiological point of view. Therefore, a future study identifying biomarkers with higher sensitivity and validity is warranted. In addition, it is necessary to examine the physiological significance of exercise-induced oxidative stress with established standard values of oxidative stress markers.

Moreover, for many years, exercise-induced oxidative stress has been interpreted with a focus on skeletal muscle based on the assumption that the production of ROS and free radicals during exercise occurs primarily in skeletal muscle. For example, several sites of ROS and free radical production in skeletal muscle have been identified with inhibitors of specific enzymes (e.g., apocynin and allopurinol) and in in vitro experiments mimicking exercise [7,22]. However, in recent years, there has been an increasing understanding that blood itself, in addition to organs including skeletal muscle, is also a source of free radical production [140]. Accordingly, it has been reported that free radicals may be produced in erythrocytes and leukocytes [141]. Therefore, the site of free radical production during exercise must be investigated through an integrative approach. In addition, sites of free radical production during exercise are speculated to be affected by experimental conditions such as exercise type (i.e., muscle contraction type), intensity, and duration. Therefore, findings on the determinants of exercise-induced oxidative stress should be accumulated step by step for each exercise condition, starting with basic research.

As a final note, it must be mentioned that exercise-induced oxidative stress has wide inter- individual variability. In a previous study, exercise-induced oxidative stress was unexpected or negligible in one in three individuals who performed high-intensity eccentric exercise (i.e., eccentric knee extension exercise) [51]. Such phenomena are also likely to be observed in treadmill or cycle ergometer exercises on a flat ground. Therefore, the large inter-individual variability in oxidative stress responses to acute exercise may provide important clues about why the study results are inconsistent. As mentioned earlier, the effects of antioxidant intake on exercise-induced oxidative stress are also reportedly affected by individual antioxidant levels at rest [124,142]. These findings indicate that when examining exercise-induced oxidative stress and how it is affected by antioxidant intake, individual redox status should be screened in advance. Moreover, further studies about the causes of wide inter-individual variability in redox status at rest are warranted.
